# Both epicardial and peri-aortic adipose tissue blunt heart rate recovery beyond body fat mass

**DOI:** 10.3389/fcvm.2022.939515

**Published:** 2022-09-21

**Authors:** Sheng-Hsiung Chang, Po-Hua Chu, Cheng-Ting Tsai, Jen-Yuan Kuo, Jui-Peng Tsai, Ta-Chuan Hung, Charles Jia-Yin Hou, Yau-Hui Lai, Chia-Yuan Liu, Wei-Ming Huang, Chun-Ho Yun, Hung-I Yeh, Chung-Lieh Hung

**Affiliations:** ^1^Division of Cardiology, Department of Internal Medicine, MacKay Memorial Hospital, Taipei, Taiwan; ^2^Department of Medicine, Mackay Medical College, New Taipei City, Taiwan; ^3^Mackay Medicine, Nursing and Management College, New Taipei City, Taiwan; ^4^Division of Cardiology, Department of Internal Medicine, MacKay Memorial Hospital, Hsinchu, Taiwan; ^5^Department of Gastroenterology, MacKay Memorial Hospital, Taipei, Taiwan; ^6^Department of Radiology, MacKay Memorial Hospital, Taipei, Taiwan

**Keywords:** epicardial adipose tissue, peri-aortic adipose tissue, multidetector computed tomography, heart rate recovery, visceral adiposity measures

## Abstract

**Background:**

Epicardial adipose tissue (EAT) as a marker of metabolic disorders has been shown to be closely associated with a variety of unfavorable cardiovascular events and cardiac arrhythmias. Data on regional-specific visceral adiposity outside the heart and its modulation on autonomic dysfunction, particularly heart rate recovery after exercise, remain obscure.

**Methods:**

We studied 156 consecutive subjects (mean age: 49.3 ± 8.0 years) who underwent annual health surveys and completed treadmill tests. Multi-detector computed tomography-based visceral adiposity, including EAT and peri-aortic fat (PAF) tissue, was quantified using dedicated software (Aquarius 3D Workstation, TeraRecon, San Mateo, CA, USA). We further correlated EAT and PAF with blood pressure and heart rate (HR) recovery information from an exercise treadmill test. Metabolic abnormalities were scored by anthropometrics in combination with biochemical data.

**Results:**

Increased EAT and PAF were both associated with a smaller reduction in systolic blood pressure during the hyperventilation stage before exercise compared to supine status (β-coefficient (coef.): −0.19 and −0.23, respectively, both *p* < 0.05). Both visceral adipose tissue mediated an inverted relationship with heart rate recovery at 3 (EAT: β-coef.: −0.3; PAF: β-coef.: −0.36) and 6 min (EAT: β-coef.: −0.32; PAF: β-coef.: −0.34) after peak exercise, even after adjusting for baseline clinical variables and body fat composition (all *p* < 0.05).

**Conclusion:**

Excessive visceral adiposity, whether proximal or distal to the heart, may modulate the autonomic response by lowering the rate of HR recovery from exercise after accounting for clinical metabolic index. Cardiac autonomic dysfunction may partly explain the increase in cardiovascular morbidity and mortality related to both visceral fats.

## Introduction

Epicardial adipose tissue (EAT) and periaortic fat (PAF) are white adipose tissue but also have highly protective functions for the adjacent myocardium and vessels through brown fat-like features (1, 2). However, both adipose tissue, estimated by multi-detector computed tomography (MDCT), have been correlated with inflammatory biochemical makers, aortic calcification, the severity of coronary artery disease (CAD), myocardial remodeling, cardiac arrhythmia, and even cardiovascular mortality (3–5). The pathogenesis of both EAT and PAF has also been associated with paracrine function. Local toxic effects of fat-related inflammatory cytokines on contacting tissues, such as coronary arteries and aorta, may promote atherogenesis and lead to an increase in adverse cardiovascular events (6, 7). Additionally, intrinsic cardiac autonomic ganglia and axons are concentrated within both EAT and PAF pads (8, 9); modulation of these autonomic ganglia can induce cardiac arrhythmia, which further the potential for cardiovascular morbidity and mortality. It has also been found that local inflammatory cytokines may influence the function of nearby nerve ganglia (10, 11). However, the exact relationship between both visceral fats and the cardiac autonomic system remains unclear. Heart rate (HR) recovery is a simple and easily available diagnostic tool that reflects the cardiac autonomic tone and has been discovered to be an important predictor of cardiovascular events (12–14). Attenuated HR recovery after exercise termination indicates either a decrease in vagal tone or an increase in sympathetic activity. In this study, we aim to evaluate the relationship between EAT, PAF, and the autonomic nervous system by using MDCT and assessing HR recovery after exercise.

## Materials and methods

### Study population

From 2005 to 2006, MDCT was performed for the assessment of coronary disease in 156 subjects who attended an annual cardiovascular health survey in a tertiary medical center in Taipei, Taiwan (Mackay Memorial Hospital). A thorough review of baseline anthropometrics and medical history was conducted by a structured questionnaire along with a detailed physical examination. The presence of cardiovascular disease history included coronary arterial disease, stroke, or prior hospitalization for heart failure. Hypertension was defined as either systolic blood pressure (SBP) of more than 140 mmHg or diastolic blood pressure (DBP) of more than 90 mmHg. Diabetes mellitus (DM) was defined as a fasting plasma glucose level of more than 126 mg/dL or a casual blood glucose level of more than 200 mg/dL. Complete body surface electrocardiograms from 12-leads and routine chest films were performed for all subjects. No subject showed significant coronary abnormality or obvious stenosis (<50%).

### Treadmill test

A standardized Bruce exercise protocol was adopted for an exercise treadmill study. HR and blood pressure during supine, standing, and standing with hyperventilation status were recorded using digitalized software. The treadmill began at 2.74 km/h (1.7 mph) at a gradient of 10%. At 3-min intervals, the incline and speed of the treadmill were increased by 2%. The maximum HR achieved was recorded and calculated as the percentage of target HR [220 beats per minute minus age in years], referenced to a value based on participant age. Participants who could not achieve more than 85% of the target HR were excluded. HR during standing and hyperventilation status were compared to that during the supine posture. We calculated the changes in HR from the peak exercise stage to those at 3- and 6-min post-exercise. SBP and DBP at 3- and 6-min post-exercise were also measured and compared to the data at peak exercise stage-derived maximal HR. All participants completed the treadmill protocol and most of the participants achieved the target heart rate and presented no strong positive treadmill results.

### Baseline anthropometric measure

All baseline characteristics and anthropometric measures, including age, body height, body weight, body mass index (BMI), and waist and buttock circumferences, were collected. The standardized sphygmomanometer cuff-defined resting blood pressure was measured under resting status by medical staff blinded to the other test results. Body fat analysis was conducted by measuring bioelectrical impedance from foot to foot, performed by a Tanita-305 body-fat analyzer (Tanita Corp., Tokyo, Japan) that provided a print-out estimate of impedance and calculation of body fat. Metabolic scores were calculated from the number of abnormal items defined by the NCEP Panel III criteria (ATP III) based on measures of waist circumference (Female ≥80 cm or Male ≥90 cm), fasting glucose (≥100 mg/dL), high-density lipoprotein cholesterol (Male <40 mg/dL or Female <50 mg/dL), triglyceride (>150 mg/dL), and blood pressure (>130/85 mmHg). A 0 score represented the absence of any abnormal metabolic components, whereas 5 represented subjects with all 5 abnormal metabolic components based on the Taiwanese population (15, 16).

### Laboratory data acquisition and analysis

A Hitachi 7170 Automatic Analyzer (Hitachi Corp. Hitachinaka Ibaraki, Japan) was used to measure glucose level (hexokinase method), total cholesterol, triglyceride, and uric acid. Lipid profiles including low- and high-density lipoprotein cholesterol (homogenous enzymatic colorimetric assay), blood urea nitrogen, and creatinine level (kinetic colorimetric assay) were obtained by a Hitachi 7170 Automatic Analyzer (Hitachi Corp. Hitachinaka Ibaraki, Japan). High-sensitivity C-reactive protein (Hs-CRP) levels were determined by highly sensitive, latex particle-enhanced immunoassay using Elecsys 2010 (Roche, Mannheim, Germany).

### Measurements of EAT and thoracic PAF by using MDCT

**Figure 1:**
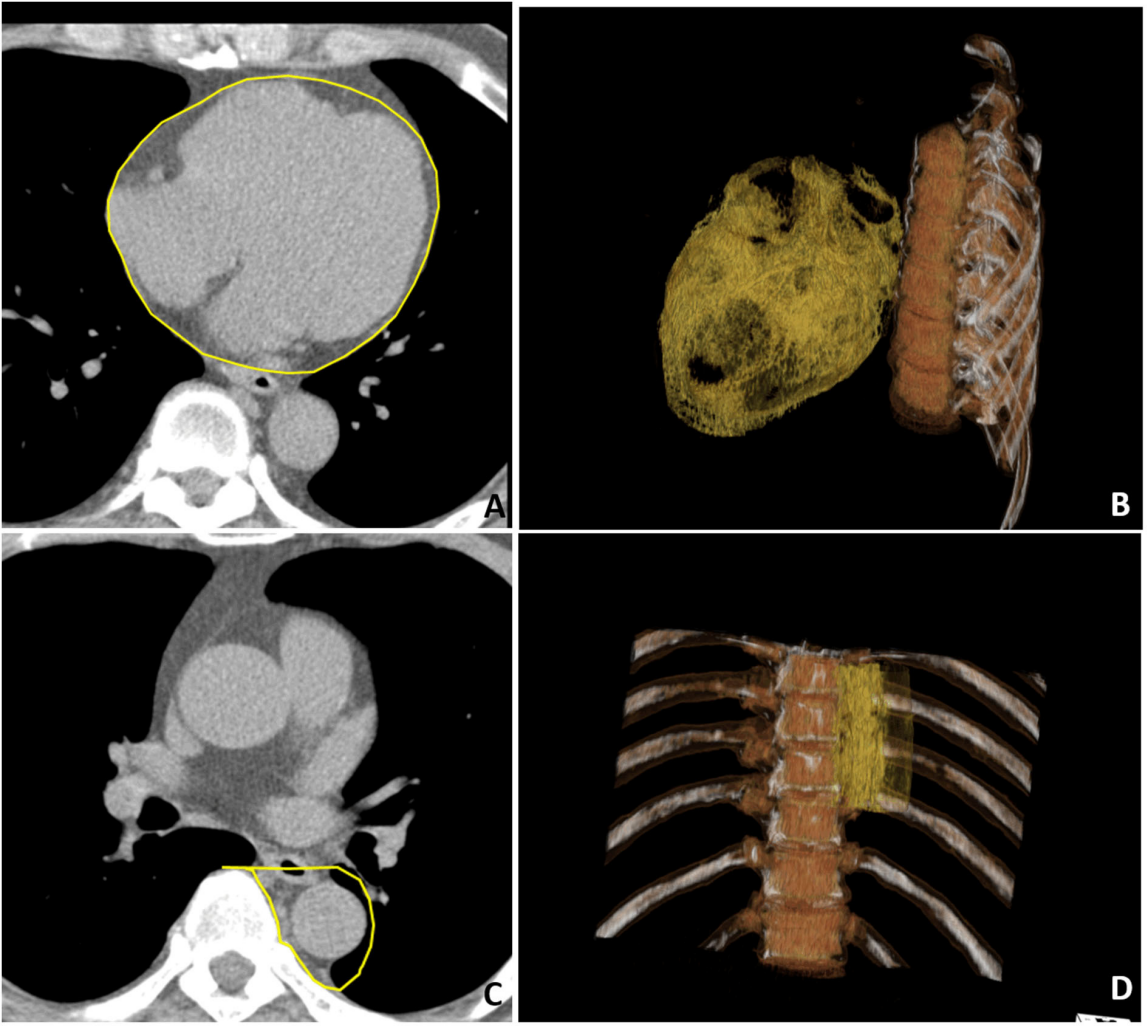
Multidetector computed tomography (MDCT) demonstrated regional-specific adiposity measures including **(A,B)** epicardial adipose tissue (EAT) and **(C,D)** peri-aortic fat (PAF) in **(A,C)** axial view and **(B,D)** three-dimensional (3D) reconstruction. **(A)** The EAT was defined as the fat inside the pericardial sac with pericardium labeled in yellow color. **(C)** The PAF was defined as the fat surrounding the thoracic descending aorta with the region of interest labeled in yellow. In the 3D reconstruction of the **(B)** EAT and **(D)** PAF, where orange regions indicate visceral fat tissue.

Cardiac computed tomography was performed by using a 16-slice MDCT scanner (Sensation 16, Siemens Medical Solutions, Forchheim, Germany) as demonstrated in a previous study (5). In brief, images were acquired from above the level of tracheal bifurcation to below the base of the heart using prospectively ECG triggering with the center of the acquisition at 70% of the R-R interval during one breath-hold period. From the raw data, the images were reconstructed with a standard kernel in 3 mm thick axial, non-overlapping slices, and a 25 cm field of view. Visceral adipose tissue of EAT and PAF was quantified by MDCT using a dedicated workstation (Aquarius 3D Workstation, TeraRecon, San Mateo, CA, USA). We traced the region of interest manually and defined fat tissue as pixels within a window of −195 to −45 Hounsfield unit (HU) and a window center of −120 HU. EAT was defined as any adipose tissue located within the pericardial sac (Figures 1A,B). Thoracic PAF was defined as the adipose tissue surrounding the thoracic aorta, which extended 67.5 mm from the level of the bifurcation of pulmonary arteries (Figures 1C,D) with cranial-caudal coverage of the thoracic aorta. Detailed methods conducted in our laboratory for similar measures have been published elsewhere (5, 17, 18).

### Statistical analysis

Continuous data were expressed as the mean and standard deviation and compared using a non-parametric trend test (Wilcoxon rank-sum test) across ordered age groups with categorical data expressed as the frequency and proportion of occurrence in all subjects. Differences in baseline demographics between groups were tested with a one-way analysis of variance (ANOVA) test with categorical data analyzed by chi-square or Fisher's exact test as appropriate. The Wilcoxon rank-sum test was used to estimate the trend of all continuous data and ordinal variables across all ordered groups. Uni- and multi-variable logistic regression models were used to determine the significant factors in the prediction of HR recovery with individual odds ratios, and significance (*p*-value) and 95% confidence intervals were reported. All data were analyzed by the commercialized software STATA 8.0 package (Stata Corp., College Station, Texas). An α-value of significance for all analyses was two-sided with 0.05 was considered to be statistically significant.

## Results

### Baseline information

Among 156 consecutively enrolled participants (mean age: 49.3±8.0 years), two of them were excluded from the assessment of the EAT because of the inadequate quality of the images. The average EAT and PAF were 75.8 ± 29.1 and 7.5 ± 3.9 ml, respectively. Tables 1, 2 list the baseline demographic data based on EAT and PAF of the participants in this study. With increasing EAT amount, the age and female gender was increasing in a significant trend (*p* < 0.001). Anthropometric data including body height, weight, body mass index, buttock or waist circumferences, and waist-to-hip ratio all increased in a significant trend across the EAT and PAF tertile groups (all trends *p* < 0.001). An ordered increase in baseline SBPs and DBPs and HRs was also observed across the tertiles (all trends *p* < 0.001). While the history of DM showed a non-significant association, hypertension increased in a significant fashion with increasing EAT and PAF in our study (both *p* < 0.01).

**Table 1:** The baseline demographic data of participants according to epicardial fat burden.

	**EAT tertile groups**			
**Variables**	**1 (*n* = 52)**	**2 (*n* = 51)**	**3 (*n* = 51)**	**Trend P**
Fat amount (ml)	<60.2	60.3 - 83.1	>83.1	
Age, years	46.6 ± 8.4	49.4 ± 7.7	52.1 ± 7.1	<0.001
Female, %	19 (36.5)	12 (23.5)	11 (22)	0.192
Height, cm	164.7 ± 8.5	166.4 ± 7.1	166.6 ± 8.1	0.410
Weight, kg	61.2 ± 9.2	68 ± 8.7	72.9 ± 11.8	<0.001
SBP, mmHg	114 ± 15.5	117.1 ± 12.6	124.6 ± 16.8	<0.001
DBP, mmHg	73 ± 11.6	75.3 ± 9.6	79.7 ± 10.5	<0.001
HR, beats/min	73.6 ± 8.6	75.9 ± 8.8	76.8 ± 11.7	0.221
Body mass index, kg/m2	22.5 ± 2.1	24.5 ± 2.4	26.2 ± 3.2	<0.001
Waist circumference, cm	77.7 ± 6.7	83.5 ± 7.4	87.8 ± 8.5	<0.001
Waist-to-hip ratio	0.86 ± 0.06	0.89 ± 0.06	0.90 ± 0.06	<0.001
Body fat composition	24.6 ± 6.1	26.2 ± 6.6	27.5 ± 6.4	0.062
HTN History, %	12 (23%)	8 (16%)	23 (45%)	0.003
DM History, %	6 (12%)	10 (20%)	8 (16%)	0.529
Fasting glucose, mg/dL	93.4 ± 10.1	98.3 ± 16.9	102.5 ± 32.7	0.144
Post-prandial glucose, mg/dL	100.6 ± 23.4	116.2 ± 42	116.7 ± 54.4	0.102
Cholesterol, mg/dL	192.9 ± 28.2	186.9 ± 29	199.3 ± 40	0.609
TG, mg/dL	114.9 ± 60.3	133.4 ± 68.1	150.3 ± 100.2	0.038
LDL-C, mg/dL	126.4 ± 28.1	121.5 ± 26.6	131.4 ± 34.1	0.671
HDL-C, mg/dL	55.5 ± 13	51.4 ± 10.9	49.2 ± 13.9	<0.001
eGFR, mL/min/1.73 m2	90.5 ± 15.8	80.1 ± 14.1	80.4 ± 12.3	0.020
Uric acid, mg/dL	5.7 ± 1.5	6.1 ± 1.6	6.0 ± 1.2	0.257
Insulin, (μU/mL)	5.02 ± 2.53	5.22 ± 2.73	6.87 ± 4.78	0.140
HOMA-IR	1.16 ± 0.65	1.3 ± 0.78	1.86 ± 1.82	0.134
HBA1C, %	5.68 ± 0.39	5.87 ± 0.37	6.14 ± 1.25	<0.001
Hs-CRP (log)	−2.89 ± 0.89	−2.68 ± 1.16	−2.4 ± 1.38	0.009
Pro-BNP (log)	3.18 ± 0.82	3.34 ± 0.75	3.31 ± 0.86	0.908

The data are presented as the mean ± SD. EAT, epicardial adipose tissue; SBP, systolic blood pressure; DBP, diastolic blood pressure; HR, heart rate; HTN, hypertension; DM, diabetes mellitus; TG, triglyceride; LDL, low-density lipoprotein; HDL, high-density lipoprotein; eGFR, estimated glomerular filtration rate; HOMA-IR, homeostatic model assessment for insulin resistance; HBA1C, glycated hemoglobin product; Hs-CRP, high-sensitivity C-reactive protein; pro-BNP, pro-hormone B-type natriuretic peptide.

**Table 2:** The baseline demographic data of participants according to peri-aortic fat burden.

	**PAF tertile groups**			
**Variables**	**1 (*n* = 53)**	**2 (*n* = 52)**	**3 (*n* = 51)**	**Trend P**
Fat amount (ml)	<5.4	5.5–8	>8	
Age, years	47.9 ± 7.9	47 ± 8.4	53.2 ± 6	<0.001
Female, %	22	44	46	<0.001
Height, cm	162.5 ± 9.1	168.1 ± 7.1	166.9 ± 6	<0.001
Weight, kg	59.4 ± 8.9	69.3 ± 8.7	72.9 ± 10.6	<0.001
SBP, mmHg	112.3 ± 16.2	119.9 ± 11.3	123.3 ± 16.5	<0.001
DBP, mmHg	71.2 ± 10.8	78.2 ± 9.6	78.6 ± 10.6	<0.001
HR, beats/min	73.4 ± 8.4	76.6 ± 10.2	76.1 ± 10.5	<0.001
Body mass index, kg/m2	22.4 ± 2.2	24.5 ± 2.3	26.1 ± 3.3	<0.001
Waist circumference, cm	76.7 ± 7.1	83.2 ± 6.3	89 ± 7.4	<0.001
Waist-to-hip ratio	0.84 ± 0.06	0.87 ± 0.04	0.92 ± 0.05	<0.001
Body fat composition	26.1 ± 6.9	25.4 ± 6.0	26.7 ± 6.3	0.981
HTN History, %	9 (17%)	12 (23%)	25 (48%)	0.003
DM History, %	6 (11%)	8 (15%)	10 (19%)	0.530
Fasting glucose, mg/dL	91.9 ± 10.4	98.9 ± 13.9	103.4 ± 33.2	0.010
Post-prandial glucose, mg/dL	112.7 ± 36.2	102.4 ± 30	119.2 ± 55.6	0.948
Cholesterol, mg/dL	189.6 ± 32.7	195.4 ± 29	194.6 ± 36.5	0.422
TG, mg/dL	97.2 ± 49.9	141.1 ± 66.9	159.1 ± 99.1	<0.001
LDL, mg/dL	121.5 ± 28.9	130.3 ± 27.7	127.7 ± 32	0.292
HDL, mg/dL	59.6 ± 14.3	49.6 ± 9.3	47.2 ± 10.7	<0.001
eGFR, mL/min/1.73 m2	89.7 ± 16.9	83.9 ± 14.4	79.1 ± 12.3	0.033
Uric acid, mg/dL	5.2 ± 1.4	6 ± 1.11	6.55 ± 1.5	<0.001
Insulin, (μU/mL)	4.14 ± 2.2	5.68 ± 2.64	7.3 ± 4.69	<0.001
HOMA-IR	0.93 ± 0.55	1.43 ± 0.82	1.98 ± 1.77	<0.001
HBA1C, %	5.67 ± 0.47	5.8 ± 0.33	6.23 ± 1.22	<0.001
Hs-CRP (log)	0.08 ± 0.06	0.11 ± 0.11	0.21 ± 0.23	<0.001
Pro-BNP (log)	41.1 ± 31.1	36 ± 52.2	37.2 ± 29.9	0.256

The data are presented as the mean ± SD. PAF, peri-aortic fat. Other abbreviations as in Table 1.

We also demonstrated the association between baseline biochemistry and PAF burden. Elevated fasting blood glucose level, insulin, homeostatic model assessment for insulin resistance, and glycated hemoglobin product were observed to correlate with higher PAF burden (all trends *p* < 0.001). In addition, both EAT and PAF volume also positively correlated with serum triglyceride (both *p* < 0.05) and showed an inverse relationship with high-density lipoprotein cholesterol level (both *p* < 0.001). Serum uric acid as well as systemic inflammatory marker Hs-CRP were observed to increase across the EAT and PAF tertiles (both *p* < 0.05).

### The relationship between standard treadmill parameters and both visceral fat amount

**Figure 2:**
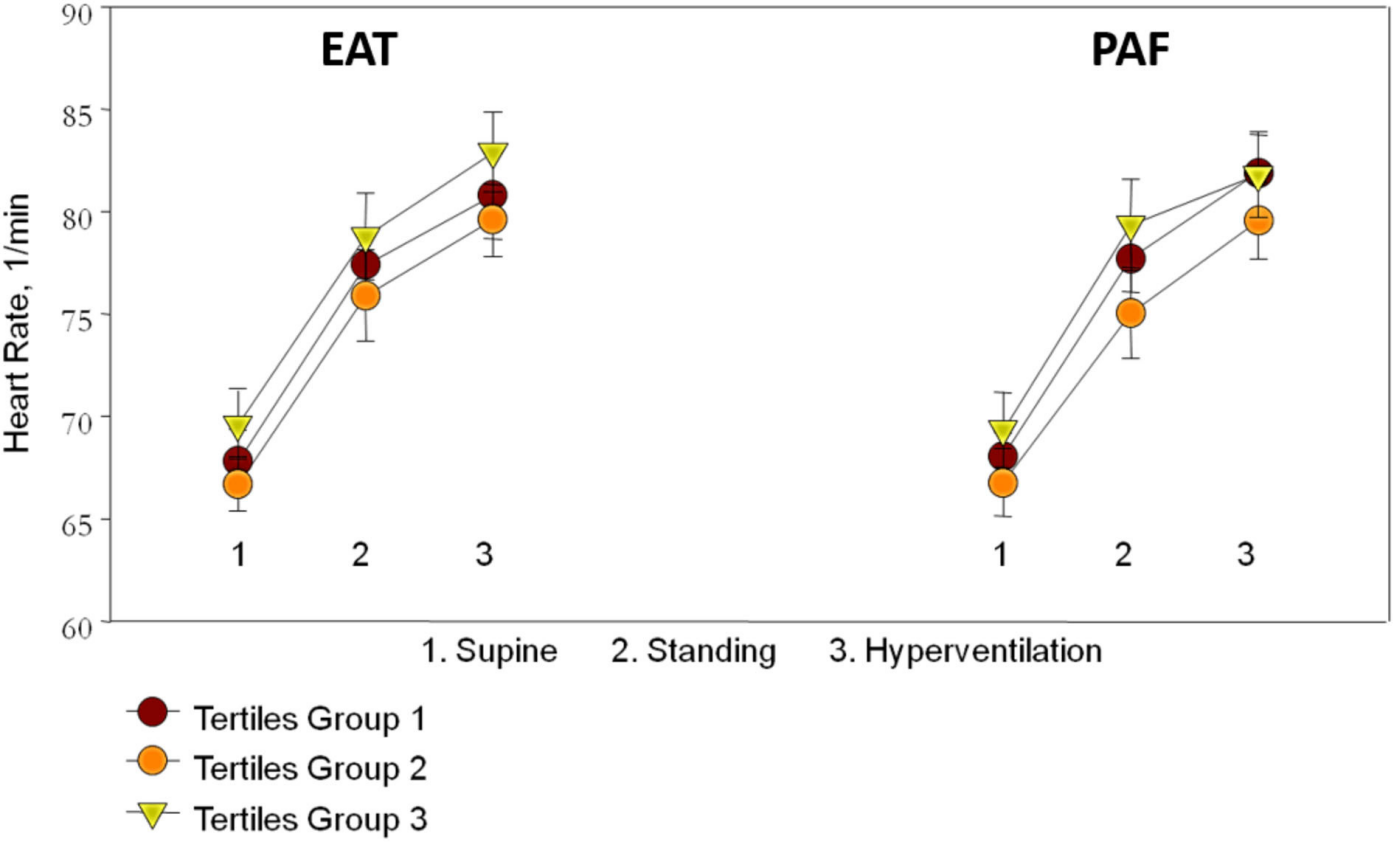
The association between resting status heart rate (HR) including sequential supine, standing postures, hyperventilation, and the tertile groups of epicardial adipose tissue (EAT) and peri-aortic fat (PAF) showed an increased trend though not reaching statistical significance.

**Figure 3:**
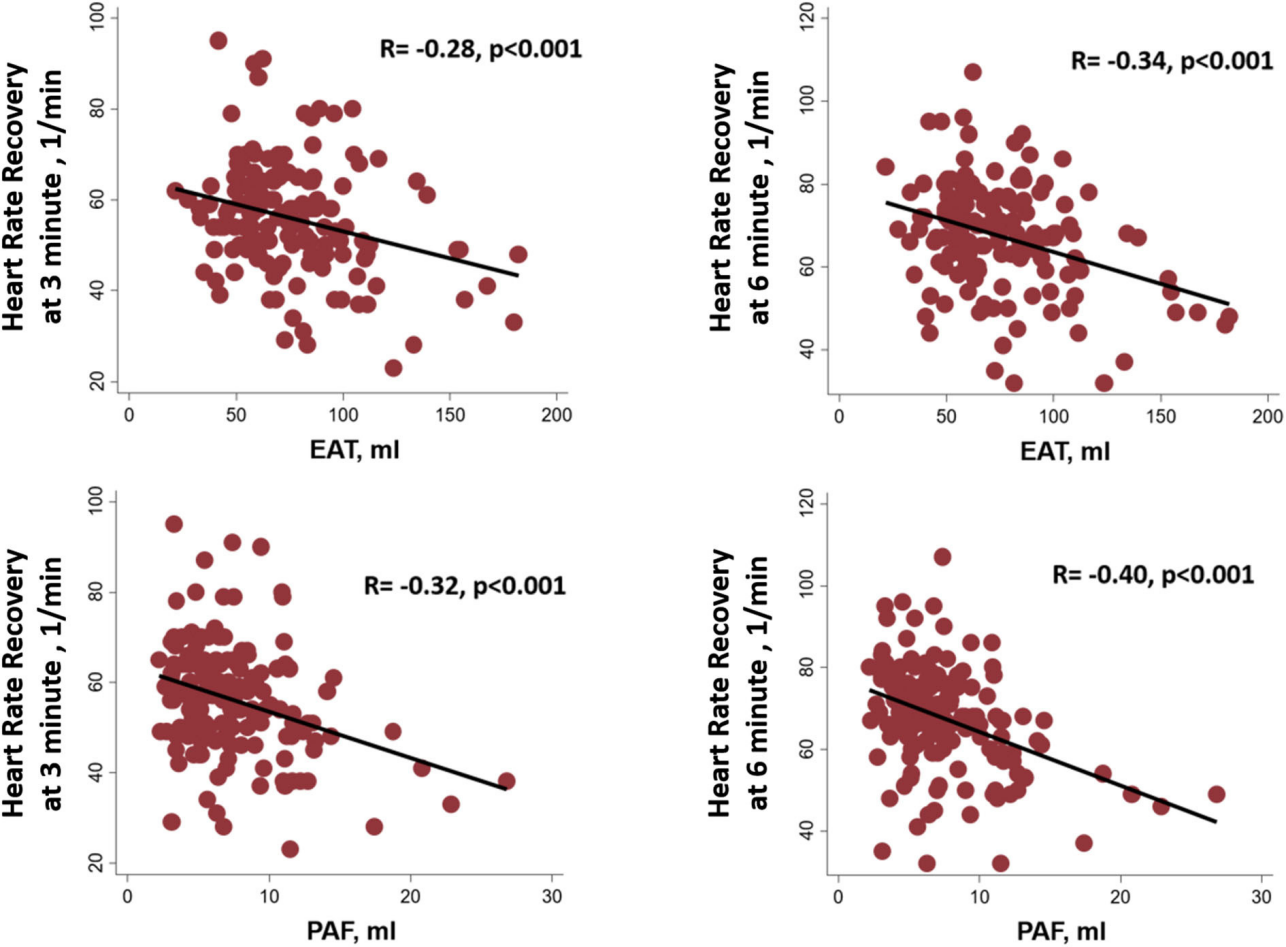
Linear relationships among the epicardial adipose tissue (EAT), peri-aortic fat (PAF), and heart rate recovery at 3 and 6 min from maximal heart rate after maximal exercise.

Resting status HRs including those taken while supine and standing as well as hyperventilation showed an increasing trend, with a general trend toward higher heart rates in the third EAT and PAF tertile groups (without statistical significance) (Figure 2). In Table 3, we showed that maximal HR was significantly reduced in the third EAT and PAF tertiles (both *p* < 0.05). In Table 4, we showed that increasing both types of visceral fats was associated with less of a reduction in SBP during the hyperventilation stage compared to that during the supine status stage (β-coefficient [coef.]: −0.23 and −0.19 for PAF and EAT, respectively, both *p* < 0.05). In addition, there were significantly inverted associations between PAF and HR increase (β-coef.: −0.2, *p*= 0.014), with a borderline inverse relationship between a larger EAT and HR increase (β-coef.: −0.15, *p* = 0.061) during the hyperventilation stage compared with that during baseline status. Finally, we also observed that both higher visceral fats were associated with significantly attenuated HR recovery at 3 (β-coef.: −0.32 and −0.28 for PAF & EAT, respectively, both *p* ≤ 0.001) and 6 min (β-coef.: −0.40 and −0.34 for PAF and EAT, respectively, both *p* < 0.001) from maximal heart rate after maximal exercise (Figure 3).

**Table 3:** The association of visceral adipose tissue and heart rate after maximum exercise.

	**1st tertile**	**2nd tertile**	**3rd tertile**	**Trend P**
**PAF**				
Maximum HR, bpm	171.96 ± 9.47	173.10 ± 17.38	165.49 ± 11.42^⋆⋇^	0.01
HR at 3 min, bpm	113.26 ± 14.55	115.98 ± 14.58	113.43 ± 14.78	0.71
HR at 6 min, bpm	101.27 ± 13.91	104.24 ± 13.14	105.25 ± 13.48	0.10
HR Recovery at 3 min	58.70 ± 11.19	57.10 ± 12.28	52.06 ± 13.04	0.003
HR Recovery at 6 min	71.41 ± 11.93	69.02 ± 13.17	61.70 ± 12.20	<0.001
**EAT**				
Maximum HR, bpm	172.79 ± 1.45	171.10 ± 2.43	166.69 ± 1.62^⋆^	0.01
HR at 3 min, bpm	114.10 ± 2.02	114.94 ± 2.07	113.84 ± 2.11	0.99
HR at 6 min, bpm	102.65 ± 1.85	103.32 ± 1.99	105.30 ± 1.98	0.27
HR Recovery at 3 min	58.69 ± 10.98	56.16 ± 12.30	52.88 ± 13.80	0.022
HR Recovery at 6 min	70.39 ± 11.22	67.53 ± 14.06	63.51 ± 13.22	0.007

The data are presented as the mean ± SD. PAF, peri-aortic fat; HR, heart rate; bpm, beats per minute; min, time interval since the termination of exercise; EAT, epicardial adipose tissue. ^⋆^ p < 0.05 (compared with 1st tertile group). ^⋇^ p < 0.05 (compared with 2nd tertile group).

**Table 4:** The regression model for visceral adipose tissue with standard treadmill parameters.

	**Uni-variable with PAF**		**Uni-variable with EAT**	
	**β-coef**.	***p*-value**	**β-coef**.	***p*-value**
HR changes as standing	0.10	0.210	0.09	0.297
HR changes as hyperventilation	−0.20	0.014	−0.15	0.061
SBP changes as standing	−0.12	0.156	−0.13	0.115
SBP changes as hyperventilation	−0.23	0.004	−0.19	0.023
DBP changes as standing	−0.09	0.284	−0.08	0.329
DBP changes as hyperventilation	−0.10	0.203	−0.03	0.740
HR recovery at 3 min	−0.32	<0.001	−0.28	0.001
HR recovery at 6 min	−0.40	<0.001	−0.34	<0.001
SBP recovery at 3 min	0.01	0.984	−0.07	0.409
SBP recovery at 6 min	0.06	0.512	−0.01	0.962
DBP recovery at 3 min	−0.01	0.936	−0.07	0.406
DBP recovery at 6 min	−0.03	0.713	−0.07	0.412

PAF, peri-aortic fat; EAT, epicardial adipose tissue; coef., coefficient; HR, heart rate; SBP, systolic blood pressure; DBP, diastolic blood pressure; min, time interval since the termination of exercise.

### Multi-variable models relating treadmill parameters and both visceral fat amount

Table 5 further demonstrated the multi-variable models with anthropometrics based on BMI between various treadmill parameters and both types of visceral fat burden. We observed that HR changes during standing and hyperventilation from resting status had borderline associations with PAF and EAT. There was a consistent inverse relationship between the visceral fat amount and HR recovery at 3 (β-coef.: −0.36 and −0.30 for PAF and EAT, respectively, both *p* < 0.05) and 6 min (β-coef.: −0.34 and −0.32 for PAF and EAT, respectively, both *p* < 0.05) after adjusting for baseline clinical variables and body fat composition.

**Table 5:** The association of visceral adipose tissue and standard treadmill parameters on multi-variable regression models.

	**PAF, ml**		**EAT, ml**		**PAF, ml**		**EAT, ml**		**PAF, ml**		**EAT, ml**	
	**Model 1**				**Model 2**				**Model 3**			
	**β-coef**.	***p***	**β-coef**.	***p***	**β-coef**.	***p***	**β-coef**.	***p***	**β-coef**.	***p***	**β-coef**.	***p***
HR changes as standing	0.20	0.072	0.20	0.070	0.20	0.075	0.22	0.048	0.23	0.092	0.32	0.012
HR changes as hyperventilation	−0.19	0.087	−0.08	0.448	−0.20	0.070	−0.09	0.403	−0.31	0.033	−0.18	0.172
SBP changes as standing	−0.09	0.480	−0.08	0.493	−0.08	0.502	−0.08	0.512	−0.06	0.698	−0.05	0.708
SBP changes as hyperventilation	−0.14	0.202	−0.09	0.431	−0.14	0.205	−0.09	0.440	0.05	0.734	0.09	0.504
DBP changes as standing	−0.11	0.357	−0.07	0.530	−0.09	0.451	−0.05	0.69	0.01	0.990	−0.01	0.968
DBP changes as hyperventilation	−0.11	0.358	0.09	0.456	−0.11	0.345	0.09	0.425	−0.12	0.278	0.29	0.033
HR recovery at 3 min	−0.39	<0.001	−0.32	0.002	−0.37	<0.001	−0.31	0.004	−0.36	0.008	−0.30	0.016
HR recovery at 6 min	−0.37	<0.001	−0.29	0.007	−0.36	0.001	−0.28	0.009	−0.34	0.010	−0.32	0.010
SBP recovery at 3 min	−0.05	0.679	−0.13	0.258	−0.05	0.689	−0.13	0.267	−0.08	0.605	−0.13	0.342
SBP recovery at 6 min	−0.08	0.511	−0.14	0.238	−0.09	0.426	−0.14	0.225	−0.27	0.069	−0.25	0.073
DBP recovery at 3 min	0.12	0.307	0.04	0.706	0.12	0.309	0.03	0.765	0.14	0.229	0.01	0.991
DBP recovery at 6 min	0.11	0.337	0.07	0.534	0.12	0.311	0.07	0.546	0.08	0.613	−0.08	0.549

Abbreviations as in Table 4.

Multivariate model 1: adjusted for age, gender, and body mass index.

Multivariate model 2: adjusted for age, gender, body mass index, hypertension, and DM history.

Multivariate model 3: adjusted for age, gender, body mass index, hypertension, DM history, serum TG, HDL, body fat, and Hs-CRP.

## Discussion

To the best of our knowledge, this was the first study to examine the association between PAF and EAT and cardiac autonomic function in terms of HR recovery from the peak exercise treadmill program. We found that both EAT and PAF volumes exert an inverted relationship with HR recovery, indicating the potential adverse effects of both visceral fats on cardiac autonomic function. The associations persisted even after adjustment for clinical variables, anthropometric data, and BMI.

### Visceral fat, cardiovascular risk factors, and cardiac autonomic function

HR is modulated by both the vagal and sympathetic nerve systems. HR recovery within a few minutes after exercise termination is a simple and easily available measure of cardiac autonomic function. The period of HR recovery after maximum exercise results from a combination of parasympathetic reactivation and sympathetic withdrawal. An attenuated HR recovery, indicating a decrease in the parasympathetic nervous system activity and/or increase in the sympathetic tone, predicts poor cardiovascular outcomes (12, 14, 19, 20). Previous studies have revealed an association between HR recovery and cardiovascular diseases, including atrial fibrillation, CAD, left ventricular diastolic dysfunction, and endothelial dysfunction (12, 21–23). The association between HR recovery and clinical cardiovascular risk factors, including hypertension, insulin resistance, dyslipidemia, and obesity, was also noted (24, 25). These conditions often coexist with increased EAT and PAF volume as shown in this and other studies (3, 5, 26–28). Previous reports have suggested that epicardial fat thickness measured on echocardiography is associated with blunted HR recovery (21, 22). However, their study groups were restricted to either men or patients with metabolic syndrome, and only epicardial fat over the free wall of the right ventricular was estimated (29, 30). Epicardial fat thickness measured using echocardiography has been suggested to provide less accurate data than epicardial fat volume measured using cardiac computed tomography (31). In this study, using a previously validated MDCT technique, we were able to estimate the total volume of both EAT and PAF. We also extended the findings by demonstrating strong correlations between both visceral fat burden and attenuated HR recovery in a cohort of the general population who answered a cardiovascular health survey even after adjustment for age, gender, BMI, waist, total body fat, cholesterol, triglyceride, and history of hypertension and DM. Our findings suggest that both EAT and PAF are more influential than systemic adiposity in cardiac autonomic function. Additionally, cardiac autonomic dysfunction, indicated by blunted HR recovery, may partly explain the increase in cardiovascular morbidity and mortality related to both visceral fats.

### Potential mechanism

Although the volume is small, EAT is a rich source of free fatty acids (FFA) because the rate of FFA synthesis and release in response to catecholamine is higher than that in other adipose tissues (32). Elevated plasma FFA level is known to alter the autonomic system by stimulating cardiac sympathetic nervous activity in both healthy subjects and DM patients (33, 34). Both EAT and PAF are also metabolically active tissues that secret cytokines and have effects on other organs through their paracrine function (6, 7). Local toxic effects of fat-related inflammatory cytokines on contact with tissues, such as coronary arteries, aorta, and myocardium, have been found to promote atherogenesis and fibrosis and lead to CAD, aortic calcification, and myocardial dysfunction (3, 5, 7). Both EAT and PAF embed intrinsic cardiac autonomic ganglia and axons, which innervate atria, ventricles, and conduction system and modulate the HR (8, 9). A previous animal study has demonstrated that interleukin-1β, an inflammatory cytokine known to increase pericardial fat (6), could modulate the excitability of ganglion neurons through a paracrine mechanism (10). Tumor necrosis factor-α, another cytokine released from pericardial fat (6), alters the cellular function of sympathetic neurons (11). Adiponectin, a protein secreted by adipocytes, can decrease the activity of autonomic ganglia (35). Thereafter, the abnormality of cardiac autonomic response manifesting reduced heart rate recovery after exercise may also result from the pathogenic effect of local inflammatory cytokines secreted from nearby visceral fats.

### Study limitations

First, this study design was cross-sectional and limited inferences of causality. Second, the study sample was comprised of Asian participants only, and thus it is uncertain if the findings may be applied to other ethnic groups. Third, we did not have complete HR recovery at 1 or 2 min after exercise in our database. However, a previous study demonstrated that a decreased HR recovery, even measured 5 min after exercise recovery, was still predictive of cardiovascular mortality (13). Our findings demonstrated that there were strong correlations between both visceral fats and HR recovery at 3 and 6 min after maximum exercise. Fourth, we did not show how the cytokines that are released by the adipose tissue are in the different tertiles. The mechanism of increased visceral adipose tissue impaired cardiac autonomic function needs further study. Finally, we do not have complete data on medications. However, the medicines that could influence the heart rate or autonomic system, e.g., beta-blockers or calcium-channel blockers, had been demonstrated to be independent of the abnormal value for HR recovery in a previous study (12). Angiotensin-converting enzyme inhibitors may also have vagomimetic activity but were proved to have no effect on HR recovery values from 3 to 10 min after maximum exercise (36). Sodium–glucose cotransporter type-2 inhibitors could potentially improve cardiac autonomic function but were not available in the period of analysis. Furthermore, we adjusted the HR recovery data for hypertension and DM, which should help reduce any potential effect modification by medications.

## Conclusion

The association between both visceral fats and blunted HR recovery was independent of traditional cardiovascular risk factors and stronger than more systemic measures of adipose tissues. Our findings showed that cardiac autonomic dysfunction, indicated by blunted HR recovery, may partly explain the increase in cardiovascular morbidity and mortality related to both visceral fats.

## Data availability statement

The raw data supporting the conclusions of this article will be made available by the authors, without undue reservation.

## Ethics statement

The studies involving human participants were reviewed and approved by Local Ethical Institutional Committee (Mackay Memorial Hospital), IRB No. 09MMHIS036. The patients/participants provided their written informed consent to participate in this study.

## Author contributions

J-YK and S-HC collected data, analyzed data, and wrote the article. C-LH, P-HC, and C-TT conceived, designed the study, and edited the article. C-LH performed the statistical analysis. J-YK and J-PT acquisition of data, analysis, and interpretation of data. T-CH, CH, Y-HL, C-YL, W-MH, and C-LH conceptual frame work. C-HY, H-IY, and C-LH reviewed article. H-IY and C-LH have given final approval to the version to be published. All authors read and approved the final manuscript.

## Funding

This research was supported by the Ministry of Science and Technology (Taiwan) (Grants NSC-101-2314-B-195-020, NSC103-2314-B-010-005-MY3, 103-2314-B-195-001-MY3, 101-2314-B-195-020–MY1, MOST 103-2314-B-195-006-MY3, NSC102-2314-B-002-046-MY3, 106-2314-B-195-008-MY2, 108-2314-B-195-018-MY2, MOST 108-2314-B-195-018-MY2, MOST 109-2314-B-715-008, and MOST 110-2314-B-715-009-MY1), MacKay Memorial Hospital (10271, 10248, 10220, 10253, 10375, 10358, and E-102003), and the Taiwan Foundation for geriatric emergency and critical care.

## Conflict of interest

The authors declare that the research was conducted in the absence of any commercial or financial relationships that could be construed as a potential conflict of interest.

## Publisher's note

All claims expressed in this article are solely those of the authors and do not necessarily represent those of their affiliated organizations, or those of the publisher, the editors and the reviewers. Any product that may be evaluated in this article, or claim that may be made by its manufacturer, is not guaranteed or endorsed by the publisher.

## References

[1] Sacks HS Fain JN Bahouth SW Ojha S Frontini A Budge H. Human epicardial fat exhibits beige features. J Clin Endocrinol Metab. (2013) 98:E1448–55. 10.1210/jc.2013-1265 23824424

[2] Vargas D Camacho J Duque J Carreño M Acero E Pérez M. Functional characterization of preadipocytes derived from human periaortic adipose tissue. Int J Endocrinol. (2017) 2017:9. 10.1155/2017/2945012 29209367 PMC5676446

[3] Rosito GA Massaro JM Hoffmann U Ruberg FL Mahabadi AA Vasan RS. Pericardial fat, visceral abdominal fat, cardiovascular disease risk factors, and vascular calcification in a community-based sample: the framingham heart study. Circulation. (2008)117:605–13. 10.1161/CIRCULATIONAHA.107.743062 18212276

[4] Cheng VY Dey D Tamarappoo B Nakazato R Gransar H Miranda-Peats R. Pericardial fat burden on ecg-gated noncontrast ct in asymptomatic patients who subsequently experience adverse cardiovascular events. JACC Cardiovasc Imaging. (2010) 3:352–60. 10.1016/j.jcmg.2009.12.013 20394896 PMC2946639

[5] Yun CH Lin TY Wu YJ Liu CC Kuo JY Yeh HI. Pericardial and thoracic peri-aortic adipose tissues contribute to systemic inflammation and calcified coronary atherosclerosis independent of body fat composition, anthropometric measures and traditional cardiovascular risks. Eur J Radiol. (2012) 81:749–56. 10.1016/j.ejrad.2011.01.035 21334840

[6] Mazurek T Zhang L Zalewski A Mannion JD Diehl JT Arafat H. Human epicardial adipose tissue is a source of inflammatory mediators. Circulation. (2003) 108:2460–6. 10.1161/01.CIR.0000099542.57313.C5 14581396

[7] Henrichot E Juge-Aubry CE Pernin A Pache JC Velebit V Dayer JM. Production of chemokines by perivascular adipose tissue: a role in the pathogenesis of atherosclerosis? Arterioscler Thromb Vasc Biol. (2005) 25:2594–9. 10.1161/01.ATV.0000188508.40052.35 16195477

[8] Singh S Johnson PI Lee RE Orfei E Lonchyna VA Sullivan HJ. Topography of cardiac ganglia in the adult human heart. J Thorac Cardiovasc Surg. (1996) 112:943–53. 10.1016/S0022-5223(96)70094-6 8873720

[9] Hou Y Scherlag BJ Lin J Zhang Y Lu Z Truong K. Ganglionated plexi modulate extrinsic cardiac autonomic nerve input: Effects on sinus rate, atrioventricular conduction, refractoriness, and inducibility of atrial fibrillation. J Am Coll Cardiol. (2007) 50:61–8. 10.1016/j.jacc.2007.02.066 17601547

[10] Takeda M Takahashi M Matsumoto S. Contribution of activated interleukin receptors in trigeminal ganglion neurons to hyperalgesia via satellite glial interleukin-1beta paracrine mechanism. Brain Behav Immun. (2008) 22:1016–23. 10.1016/j.bbi.2008.03.004 18440198

[11] Soliven B Albert J. Tumor necrosis factor modulates Ca^2+^ currents in cultured sympathetic neurons. J Neurosci. (1992) 12:2665–71. 10.1523/JNEUROSCI.12-07-02665.1992 1377234 PMC6575834

[12] Cole CR Blackstone EH Pashkow FJ Snader CE Lauer MS. Heart-rate recovery immediately after exercise as a predictor of mortality. N Engl J Med. (1999) 341:1351–7. 10.1056/NEJM199910283411804 10536127

[13] Cheng YJ Lauer MS Earnest CP Church TS Kampert JB Gibbons LW. Heart rate recovery following maximal exercise testing as a predictor of cardiovascular disease and all-cause mortality in men with diabetes. Diabetes Care. (2003) 26:2052–7. 10.2337/diacare.26.7.2052 12832312

[14] Vivekananthan DP Blackstone EH Pothier CE Lauer MS. Heart rate recovery after exercise is a predictor of mortality, independent of the angiographic severity of coronary disease. J Am Coll Cardiol. (2003) 42:831–8. 10.1016/s0735-1097(03)00833-7 12957428

[15] Alberti KG Eckel RH Grundy SM Zimmet PZ Cleeman JI Donato KA. Harmonizing the metabolic syndrome: a joint interim statement of the international diabetes federation task force on epidemiology and prevention; national heart, lung, and blood institute; american heart association; world heart federation; international atherosclerosis society; and international association for the study of obesity. Circulation. (2009) 120:1640–5. 10.1161/CIRCULATIONAHA.109.192644 19805654

[16] Fan JG Saibara T Chitturi S Kim BI Sung JJ Chutaputti A. What are the risk factors and settings for non-alcoholic fatty liver disease in Asia-Pacific. J Gastroenterol Hepatol. (2007) 22:794–800. 10.1111/j.1440-1746.2007.04952.x 17498218

[17] Sung KT Kuo R Sun JY Hung TC Chang SC Liu CC. Associations between CT-determined visceral fat burden, hepatic steatosis, circulating white blood cell counts and neutrophil-to-lymphocyte ratio. PLoS ONE. (2018) 13:e0207284. 10.1371/journal.pone.0207284 30458019 PMC6245737

[18] Yang FS Yun CH Wu TH Hsieh YC Bezerra HG Liu CC. High pericardial and peri-aortic adipose tissue burden in pre-diabetic and diabetic subjects. BMC Cardiovasc Disord. (2013) 13:98. 10.1186/1471-2261-13-98 24499326 PMC3832227

[19] Arena R Guazzi M Myers J Peberdy MA. Prognostic value of heart rate recovery in patients with heart failure. Am Heart J. (2006) 151:851–13. 10.1016/j.ahj.2005.09.012 16569547

[20] Kannankeril PJ Le FK Kadish AH Goldberger JJ. Parasympathetic effects on heart rate recovery after exercise. J Investig Med. (2004) 52:394–401. 10.1136/jim-52-06-34 15612453

[21] Gera N Taillon LA Ward RP. Usefulness of abnormal heart rate recovery on exercise stress testing to predict high-risk findings on single-photon emission computed tomography myocardial perfusion imaging in men. Am J Cardiol. (2009) 103:611–4. 10.1016/j.amjcard.2008.11.004 19231321

[22] Maddox TM Ross C Ho PM Magid D Rumsfeld JS. Impaired heart rate recovery is associated with new-onset atrial fibrillation: a prospective cohort study. BMC Cardiovasc Disord. (2009) 9:11. 10.1186/1471-2261-9-11 19284627 PMC2660286

[23] Huang PH Leu HB Chen JW Cheng CM Huang CY Tuan TC. Usefulness of attenuated heart rate recovery immediately after exercise to predict endothelial dysfunction in patients with suspected coronary artery disease. Am J Cardiol. (2004) 93:10–3. 10.1016/j.amjcard.2003.09.004 14697458

[24] Spies C Otte C Kanaya A Pipkin SS Schiller NB Whooley MA. Association of metabolic syndrome with exercise capacity and heart rate recovery in patients with coronary heart disease in the heart and soul study. Am J Cardiol. (2005) 95:1175–9. 10.1016/j.amjcard.2005.01.045 15877989 PMC2776681

[25] Yeckel CW Gulanski B Zgorski ML Dziura J Parish R Sherwin RS. Simple exercise recovery index for sympathetic overactivity is linked to insulin resistance. Med Sci Sports Exerc. (2009) 41:505–15. 10.1249/mss.0b013e31818afa2f 19204601

[26] Al Chekakie MO Welles CC Metoyer R Ibrahim A Shapira AR Cytron J. Pericardial fat is independently associated with human atrial fibrillation. J Am Coll Cardiol. (2010) 56:784–8. 10.1016/j.jacc.2010.03.071 20797492

[27] Aydin H Toprak A Deyneli O Yazici D Tarcin O Sancak S. Epicardial fat tissue thickness correlates with endothelial dysfunction and other cardiovascular risk factors in patients with metabolic syndrome. Metab Syndr Relat Disord. (2010) 8:229–34. 10.1089/met.2009.0080 20156077

[28] Iacobellis G Leonetti F Singh N. Relationship of epicardial adipose tissue with atrial dimensions and diastolic function in morbidly obese subjects. Int J Cardiol. (2007) 115:272–3. 10.1016/j.ijcard.2006.04.016 16759715

[29] Kim MK Tanaka K Kim MJ Matsuo T Tomita T Ohkubo H. Epicardial fat tissue: Relationship with cardiorespiratory fitness in men. Med Sci Sports Exerc. (2010) 42:463–9. 10.1249/mss.0b013e3181b8b1f0 19952810

[30] Sengul C Duman D. The association of epicardial fat thickness with blunted heart rate recovery in patients with metabolic syndrome. Tohoku J Exp Med. (2011) 224:257–62. doi.org/10.1620/tjem.224.257 21737994 10.1620/tjem.224.257

[31] Greif M Becker A von Ziegler F Lebherz C Lehrke M Broedl UC. Pericardial adipose tissue determined by dual source ct is a risk factor for coronary atherosclerosis. Arterioscler Thromb Vasc Biol. (2009) 29:781–6. 10.1161/ATVBAHA.108.180653 19229071

[32] Marchington JM Mattacks CA Pond CM. Adipose tissue in the mammalian heart and pericardium: Structure, foetal development and biochemical properties. Comp Biochem Physiol B. (1989) 94:225–32. 10.1016/0305-0491(89)90337-4 2591189

[33] Paolisso G Manzella D Rizzo MR Ragno E Barbieri M Varricchio G. Elevated plasma fatty acid concentrations stimulate the cardiac autonomic nervous system in healthy subjects. Am J Clin Nutr. (2000) 72:723–30. 10.1093/ajcn/72.3.723 10966890

[34] Manzella D Barbieri M Rizzo MR Ragno E Passariello N Gambardella A. Role of free fatty acids on cardiac autonomic nervous system in noninsulin-dependent diabetic patients: Effects of metabolic control. J Clin Endocrinol Metab. (2001) 86:2769–74. 10.1210/jcem.86.6.7553 11397885

[35] Zhou Z Li SY Sheng X Liu ZH Lai YQ Wang ML. Interactions between metabolism regulator adiponectin and intrinsic cardiac autonomic nervous system: a potential treatment target for atrial fibrillation. Int J Cardiol. (2020) 302:59–66. 10.1016/j.ijcard.2019.12.031 31889562

[36] Ilke Sipahi Goknur Tekin Zerrin Yigit Deniz Guzelsoy Ozen Guven. Effect of quinapril on the attenuated heart rate recovery of type 2 diabetic subjects without known coronary artery disease. Clin Cardiol. (2004) 27:480–4 10.1002/clc.4960270812 PMC6654683 15346847

